# Skeletal Muscle Disease: Imaging Findings Simplified

**DOI:** 10.7759/cureus.29655

**Published:** 2022-09-27

**Authors:** Daphne J Theodorou, Stavroula J Theodorou, Luca Saba, Yousuke Kakitsubata

**Affiliations:** 1 Radiology, MRI-CT Unit, General Hospital of Ioannina, Ioannina, GRC; 2 Radiology, Musculoskeletal MRI, University Hospital of Ioannina, Ioannina, GRC; 3 Radiology, Azienda Ospedaliero Universitaria di Cagliari, Cagliari, ITA; 4 Radiology, Miyazaki Konan Hospital, Miyazaki, JPN

**Keywords:** fat, mass, edema, disease, patterns, mri, myopathy, muscle disease, muscle

## Abstract

Skeletal muscle is a major anatomic structural component of the human body. Myopathy, defined as skeletal muscle disease, may offend any of the body’s 650 muscles and encompasses an extended array of acute and chronic abnormalities. Muscle disease can be categorized according to etiology as congenital, traumatic, infectious, or neoplastic. The concept of the diversity of multiple muscular disease processes signifies an important role for imaging in the detection and characterization of myopathy. However, despite the exquisite physiological properties of skeletal muscle, muscle imaging has not received attention equal to that of bones and joints. Accordingly, this article provides an indication of the most suitable imaging modalities for myopathy and reviews a multitude of primary and systemic muscle derangements, with an emphasis on magnetic resonance (MR) imaging findings. Because these patterns of MR imaging abnormality bespeak the widespread nature of myopathy, we illustrate typical examples of muscle disease processes to simplify diagnosis.

## Introduction and background

Skeletal muscle, measuring up to 60% of the total protein stores, comprises the most abundant tissue in the human body of young adults [1,2]. Skeletal striated muscle, also referred to by its Latin name, muscularis striatus skeletalis, is a complex organ, which contains multiple bundles of cells known as muscle fibers. Muscle fibers comprising the basic structural element of skeletal muscle are cylindrical and are composed of myofibrils, which, in turn, are composed of the contractile proteins actin and myosin. As a major functional unit surrounding bones, skeletal muscles orchestrate all coordinated body movements that enable our activities of daily living, and, as such, any offense in muscle mass can be responsible for considerable disability. Because patients with muscle derangements may present with non-specific symptoms of myalgia (pain), weakness, and fatigue, abnormalities in muscle are often overlooked or underestimated as a source of pathology. Not infrequently, clinical assessment of the integrity and performance of muscle is difficult owing to complex compartmental anatomy and numerous anatomic variations, making a presumptive diagnosis of skeletal muscle disease rely strongly on a clinical imaging correlation. Despite advanced imaging methods for muscle disease, biopsy remains the cornerstone of diagnosis that proves valuable in challenging, or indeterminate, cases [3-6].

Myopathy is associated with an infinite variety of infectious, inflammatory, traumatic, neurological, genetic, neoplastic, and iatrogenic conditions that can cause pain and disability, and, as such, specific imaging is required [3-5]. Although diverse, some diseases offending muscle share similar imaging appearances, whereas others present distinct patterns of imaging abnormality. Magnetic resonance (MR) imaging is well suited for the direct and detailed assessment of soft tissue, including muscle. Key MR imaging findings of myopathy generally fall into one of three cardinal patterns: muscle edema, fatty infiltration, and mass lesion [6]. To address the clinical heterogeneity of muscle abnormalities, we revisit the fundamental MR signal alterations associated with common and uncommon muscular derangements. Importantly, this article aims to provide clinicians with a succinct and practical imaging guide for diagnosing myopathy.

## Review

Imaging in myopathy

A summary of the imaging techniques used in the analysis of several primary and systemic disorders affecting skeletal muscle is provided in Table 1.

**Table 1:** Imaging assessment of myopathy. CT: computed tomography; MRI: magnetic resonance imaging

Modality	Pros	Cons
Radiography	Initial assessment of suspected abnormality	Suboptimal for deep-seated or nonmineralized lesions
	Alerts for further imaging	Unrewarding if complex anatomy
Ultrasound	Evaluation of superficial soft-tissue masses	Operator dependent
	Differentiation of solid from cystic lesions	Limited reproduction of images
	Evaluation of muscle size and echogenicity	
	Assessment of musculotendinous junction integrity	
	Simultaneous assessment of neurovascular structures	
CT	Assessment of muscle morphology and attenuation	Limited contrast resolution for muscle
	Characterization of soft-tissue mineralization	Use of ionizing radiation
	Guides percutaneous soft-tissue biopsy	
MRI	Evaluation of muscle morphology and intrinsic characteristics	Less sensitive for soft-tissue mineralization
	Detection of muscle edema, fatty infiltration, mass lesion	Susceptible to motion artifacts, pacemakers
	Assessment of bone marrow and neurovascular structures	

Following a brief review of the imaging studies used to investigate muscular disease, we focus on the diagnosis of myopathy by MR imaging.

Radiography

Radiography may reveal indirect signs of muscle abnormalities including an increase in soft-tissue volume or obliteration of the fat planes. Furthermore, radiography allows for the evaluation of muscle derangements characterized by the abnormal deposition of radiodense material in the form of calcification or ossification in muscle or musculotendinous structures (i.e., calcium hydroxyapatite crystal deposition, phleboliths, heterotopic ossification), formation of gas (necrotizing myositis/fasciitis), retention of metallic foreign bodies (penetrating soft-tissue injuries), and deposition of fat (lipoma). Although radiography is characterized by an overall lack of anatomical detail and poor sensitivity, it often serves as a guide for further imaging [7].

Sonography

Sonography proves helpful in the setting of a suspected superficial lesion and finds use in the differentiation between solid and cystic lesions in muscle, or the discrimination of a mass lesion from diffuse edema [8]. With high-frequency transducers, the normal muscle appears heterogeneous, with linear areas of bright echotexture representing fat interspersed among hypoechoic muscle fibers. Tendons and the musculotendinous junction typically are hyperechoic structures. Attention is needed not to overcall tendinosis or tendon tear due to anisotropy artifact that may be seen on the sonography images. The additional application of functional duplex or color Doppler imaging permits real-time investigation of soft-tissue vascularity, granting sonography the advantage to provide details about the relationships between a given lesion and adjacent neurovascular structures [9]. Despite being operator-dependent, sonography is widely accessible, portable, safe, and low cost, and can be efficaciously used for imaging in children and emergencies.

Computed tomography

CT provides important clinical information in the evaluation of areas of complex anatomy that may harbor muscle pathology. Although CT is generally characterized by limited contrast resolution for muscle, it can assess muscle morphology and size and may delineate muscle replacement by fat [10]. CT has a useful role in the depiction of mineralized matrix and the detection of gas within the soft tissue and can guide percutaneous soft-tissue biopsy. CT is a quick, relatively inexpensive, and almost universally available imaging technique. A major limitation of CT involves exposure of the patient to ionizing radiation [4,5].

Magnetic resonance imaging

MR imaging is the imaging method of choice for diagnosing skeletal muscle disease, providing excellent soft-tissue contrast resolution and multiplanar tomographic display [5]. Although MR imaging is less sensitive than CT in the evaluation of soft-tissue mineralization, it can readily assess muscle size, shape, and signal intensity changes with no ionizing radiation. Furthermore, MR imaging can depict selective abnormality within individual muscles that may be challenging to detect clinically because of the presence of unaffected synergistic muscles [11-13]. Prime indications for MR imaging of muscle include (i) characterization of athletic injuries and estimate of time to recovery; (ii) investigation of soft-tissue mass and vascularity; (iii) documentation of the presence, extent, and other manifestations of infection; and (iv) assessment of the presence of myopathy with or without neuropathy, particularly when MR imaging can guide preoperative planning, or evaluate response to treatment. It should be emphasized, however, that MR imaging in the appropriate clinical setting may help in limiting the differential diagnosis, selecting new therapeutic regimens, monitoring disease, and assessing response to treatment or complications in patients with variable muscle insults. Limitations of MR imaging include cost, availability, incompatible foreign materials, artifacts, and limited access for severely ill or claustrophobic patients.

Vast technological advances have taken place in the field of radiology led by the introduction of MR imaging with several quantitative, dynamic, and functional techniques developed for studying the composition, architecture, and mechanical properties of muscle [13-15]. Chemical shift imaging (in-phase and opposed-phase imaging) allows for fat quantification in neuromuscular disorders including Duchenne muscular dystrophy, and the distinction of soft-tissue tumors from reactive marrow changes. Diffusion-weighted MR imaging (DWI) may allow for advanced qualitative assessment of inflammatory myopathies, or the differentiation of benign from malignant lesions. Diffusion tensor imaging (DTI) has been utilized for studying skeletal muscle microarchitecture via fiber tracking which enables the detection of subclinical injury associated with trauma, ischemia, inflammation, or neuropathy. MR spectroscopy may provide information about muscle composition and metabolism and has been used to assess muscular dystrophies. Dynamic-phase contrast imaging allows for real-time imaging and has been used in biomechanical analyses of muscle contraction and fibrosis. Stimulus MRI utilizes electrical stimuli to study micro-changes in muscular tissue. MR imaging elastography has been applied to study the mechanical properties of muscle, including assessment of stiffness, temperature, and morphology. Functional MRI, including skeletal muscle perfusion imaging, has been used to study muscular microcirculation in sports medicine, detect overuse injuries, and the pharmacological effects of specific medications (i.e., vasodilators). T2 relaxation time mapping offers potential for characterizing metabolic activity in muscle, especially immediately after exercise or in the context of pediatric muscular dystrophies. Whole-body MRI utilizes a wide field of view to simultaneously image entire body musculature at a glance to characterize patterns of generalized myopathy. However, because many of the above-mentioned advanced MR imaging techniques have been used for research purposes, the results derived from these studies are not currently validated in large series of patients and therefore should not be considered a standard diagnostic imaging tool [13].

Major patterns of muscle involvement: the basics

Despite dramatic changes that have been realized with continued improvements in imaging technology and the development of new MR imaging protocols, diagnosis of myopathy can be particularly challenging. However, the fundamental principles of the assessment of muscle derangements have remained constant, and are summarized into three broad categories based on the presence of edema, fatty infiltration, and mass.

Muscle Edema Pattern

**Figure 1:**
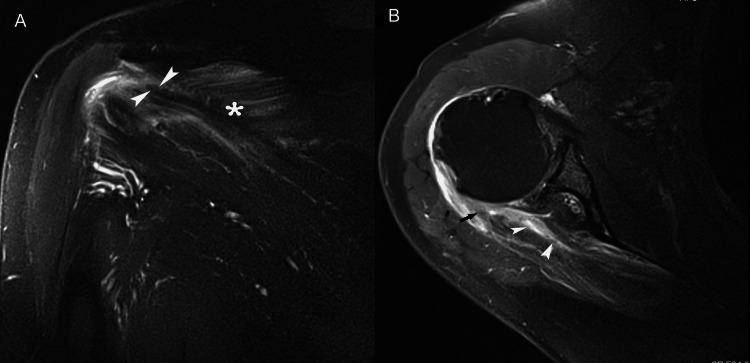
A 23-year-old man presented with pain in the right shoulder after an intense weight-lifting workout. (A) Coronal short-tau inversion recovery image shows abnormal high signal intensity in infraspinatus muscle, without obvious architectural distortion of muscle. Edema in a “feathery” pattern is tracking along muscle fascicles (asterisk) and around epimysium (arrowheads) between cranial and central parts of the infraspinatus. (B) Axial fat-suppressed fast spin-echo T2-weighted MR image reveals hyperintense fluid within the muscle belly (arrowheads), consistent with a strain of the infraspinatus muscle. Note extensive edema at the myotendinous junction (arrow) of the torn infraspinatus tendon.

**Figure 2:**
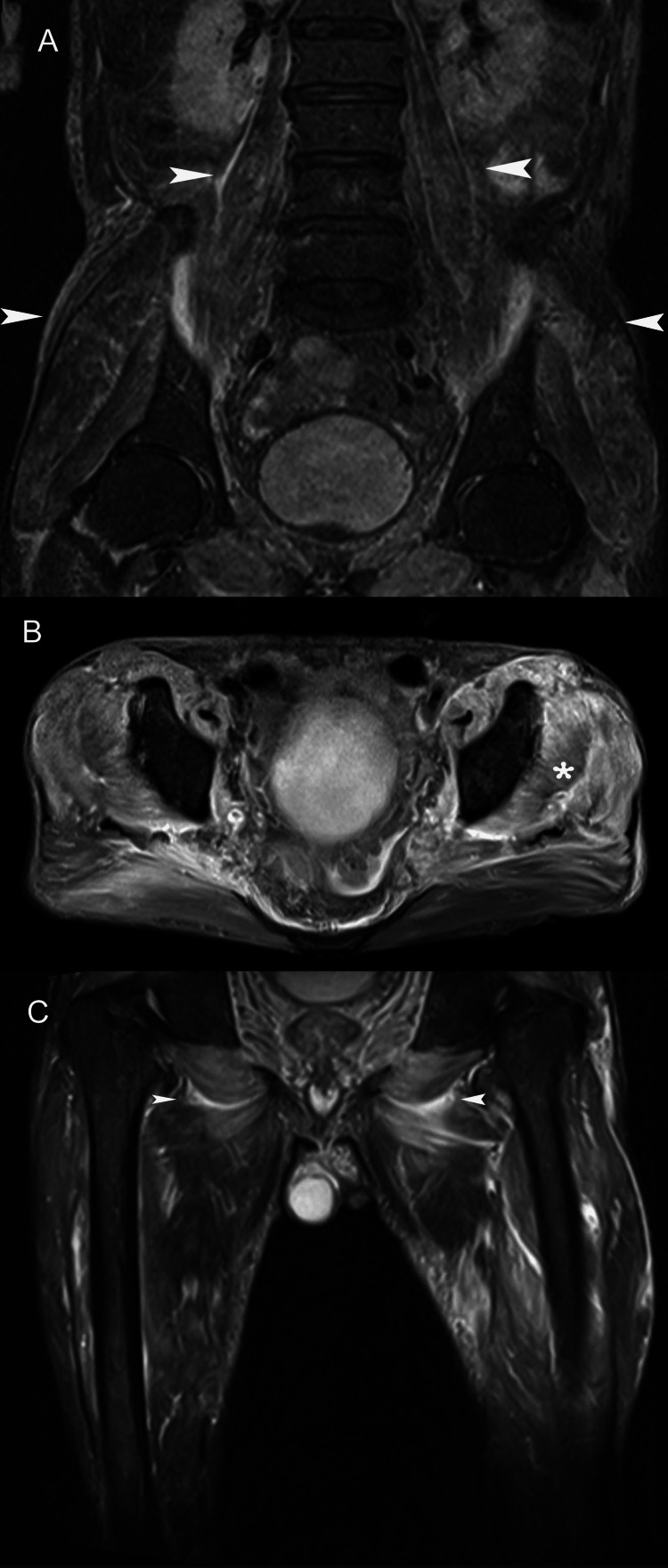
A 52-year-old man presented with weakness, malaise, and painful swelling of the trunk and thighs. Muscle biopsy disclosed nonspecific (idiopathic) myositis, and the patient was started on steroids with rapid clinical improvement. (A) Coronal fat-suppressed fast spin-echo T2-weighted MR image shows diffuse edema in psoas muscles and pelvic girdle musculature (arrowheads). (B) Axial short-tau inversion recovery MR image through the pelvis shows diffuse increased signal in enlarged gluteal muscles (asterisk). (C) Coronal fat-suppressed fast spin-echo T2-weighted MR image shows diffuse edema in the adductor magnus and peritrochanteric muscles.  Perifascial fluid is seen between the obturator internus and externus muscles (arrowheads).

**Figure 3:**
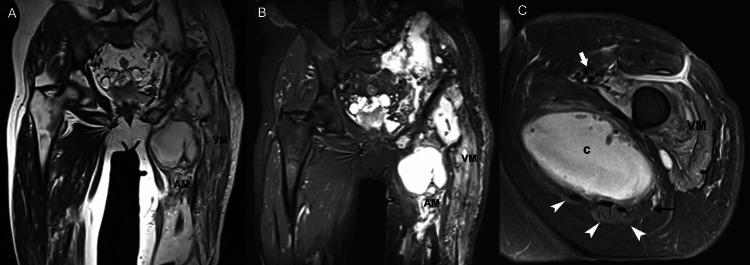
A 65-year-old man with generalized bone and soft-tissue echinococcosis of the left hemipelvis and the thigh. Coronal T2-weighted (A) and short-tau inversion recovery (B) images show diffuse edema in the vastus intermedius and lateralis muscles (VM) coupled with marked atrophy of the adductor (AM) muscles, in contrast with the normal appearance of the opposite side. (C) Axial short-tau inversion recovery image through the thigh shows the denervation of the vastus muscles (VM) owing to the inflammation of the femoral nerve (thick arrow). A large parasitic cyst (c) growing within the adductor magnus muscle causes compression of the sciatic nerve (arrow), and atrophy of muscles in the posterior compartment of the thigh (arrowheads).

**Figure 4:**
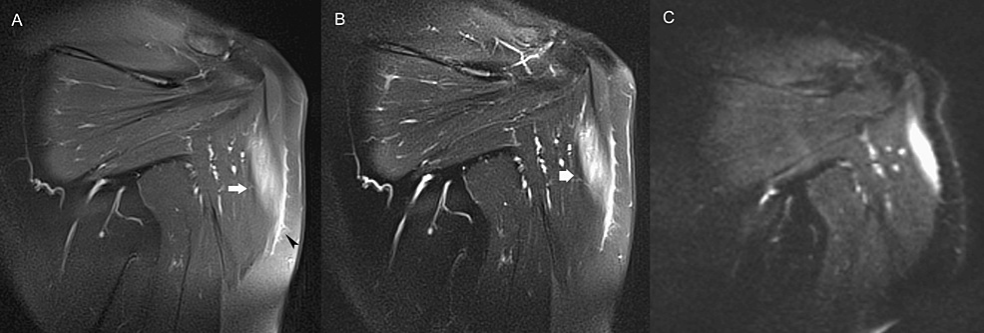
COVID-19 vaccine-related myositis in a middle-aged woman. (A) Coronal T2-weighted MR image with fat saturation shows edema infiltrating the deltoid muscle (arrow). Note the perifascial fluid (arrowhead). (B) Coronal T1-weighted MR image with fat saturation after gadolinium administration shows intense enhancement of inflamed deltoid muscle (thick arrow). (C) Coronal diffusion-weighted image (b-value 800 s/mm^2^) shows increased diffusion in the deltoid muscle, representing increased capillary perfusion due to active inflammation.

Acute or recent insult to muscles is characterized by edema, vascular engorgement, and inflammatory cellular infiltration [5]. On MR images, pathologic changes corresponding to edema are manifest as areas of low-to-intermediate signal intensity on T1-weighted images and high signal intensity on T2-weighted and inversion-recovery images. Many abnormalities can substantiate a muscle edema pattern, including traumatic injury (i.e., strain, contusion), muscular exertion (i.e., acute muscle soreness, delayed-onset muscle soreness, DOMS), rhabdomyolysis, myonecrosis, vascular insults (i.e., compartment syndrome, diabetic infarction), early myositis ossificans, subacute denervation, radiation therapy, and myositis (i.e., autoimmune, idiopathic, infectious, COVID-19 vaccine-related, sarcoid myopathy) [3,7,16-20] (Figures 1-4).

Advanced imaging including MR spectroscopy, T2 imaging, diffusion-weighted, diffusion tensor, and perfusion imaging has been used to assess complex changes in the amount and distribution of intracellular, extracellular, and mitochondrial water protons in skeletal muscle. Clinical information of preceding trauma, myoglobinuria, elevated intracompartmental pressure, signs of infection, and motosensory deficits are significant confounding factors in diagnosis.

Fatty Infiltration Pattern

**Figure 5:**
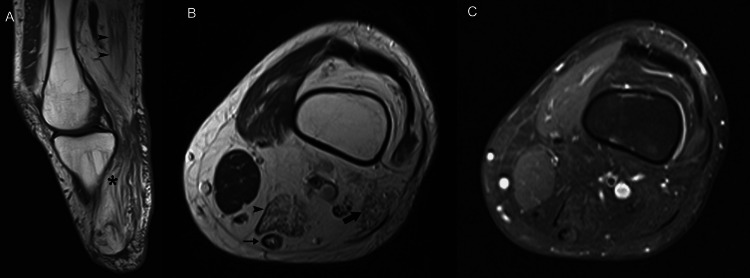
A 48-year-old man after remote below-knee amputation. (A) Sagittal T1-weighted MR image shows increased signal consistent with fatty infiltration of atrophied semimembranosus (arrowheads) and gastrocnemius muscles (asterisk) due to chronic disuse. (B) Axial T2-weighted MR image shows fatty infiltration pattern involving semimembranosus (arrowhead), semitendinosus (arrow), and biceps femoris (thick arrow) muscles. (C) Corresponding T2-weighted MR image with fat suppression reveals a lack of high-signal-intensity fat within infiltrated muscles.

**Figure 6:**
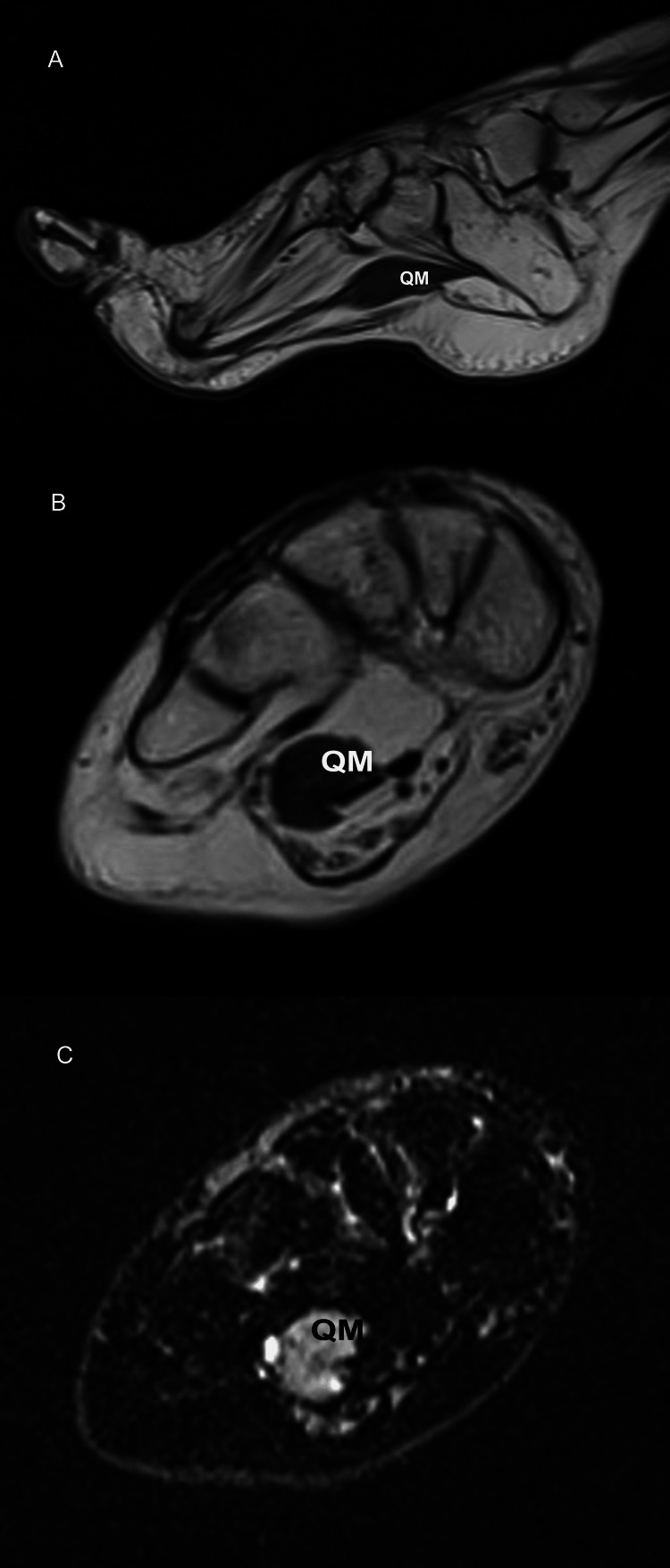
A 46-year-old female refugee with foot drop related to skeletal muscle channelopathy (sodium channel myotonia). (A) Sagittal T1-weighted MR image shows pes cavus deformity with severe, gross fatty atrophy of intrinsic foot muscles. The quadratus plantae muscle (QM) is spared. Axial T2-weighted (B) and short-tau inversion recovery (C) MR images show QM of intermediate T2-weighted and predominant high signal intensity on short-tau inversion recovery images. Abnormal increased inward flux of sodium in muscle cells may account for edema-like changes in the quadratus plantae muscle.

**Figure 7:**
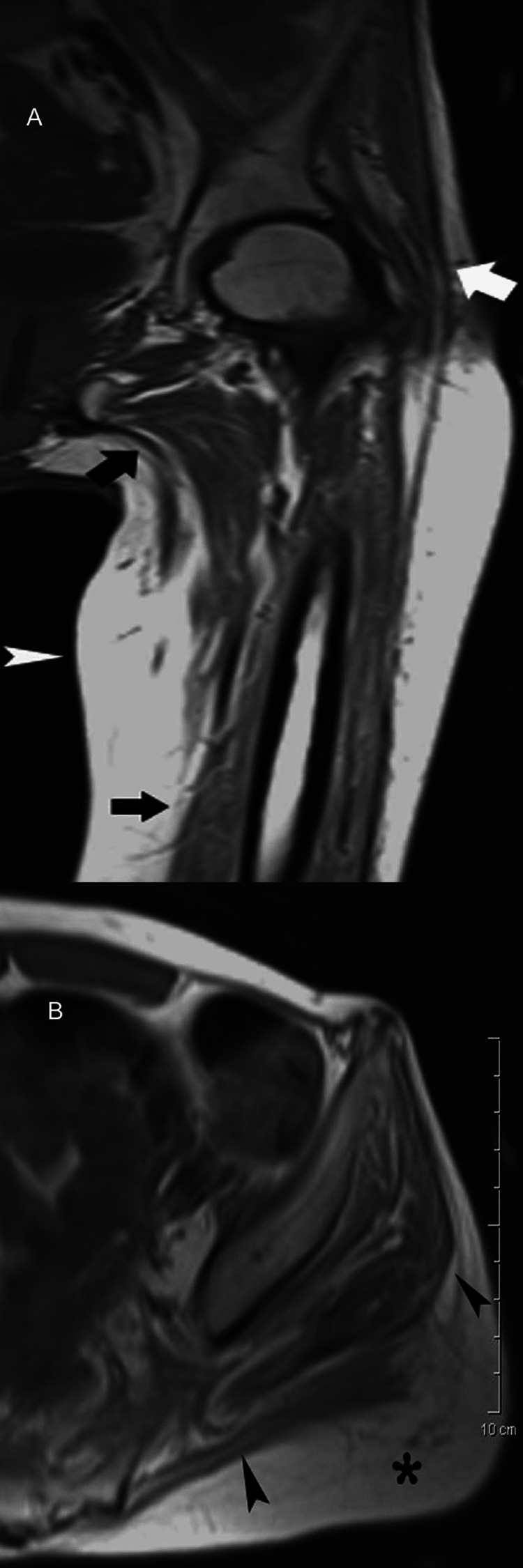
A 34-year-old male with muscle weakness after receiving prolonged treatment with corticosteroids for Crohn’s disease. (A) Coronal T1-weighted MR image reveals overt generalized atrophy of muscles around the hip and thigh (arrows). Note the increased accumulation of fat in the thigh (arrowhead). (B) Corresponding axial T1-weighted MR image shows marked fatty infiltration of the gluteal muscles (arrowheads). The regional fat depot is seen in the buttock (asterisk).

Non-acute or chronic muscle insult may result in dystrophic changes with abnormal fatty infiltration and muscular atrophy. Progressive changes are characterized by the presence of fat signal intensity on both T1-weighted and T2-weighted images. Among the conditions associated with fatty degeneration of muscle are chronic stages of muscle injury or musculotendinous injury (i.e., tendon tear), chronic disuse, denervation, hereditary or acquired myopathies (myotonic dystrophy, skeletal muscle channelopathies, collagen muscular dystrophy, inclusion body myositis), use of corticosteroids, lipomatous lesions, and normal aging [3,11,21] (Figures 5-7).

**Figure 8:**
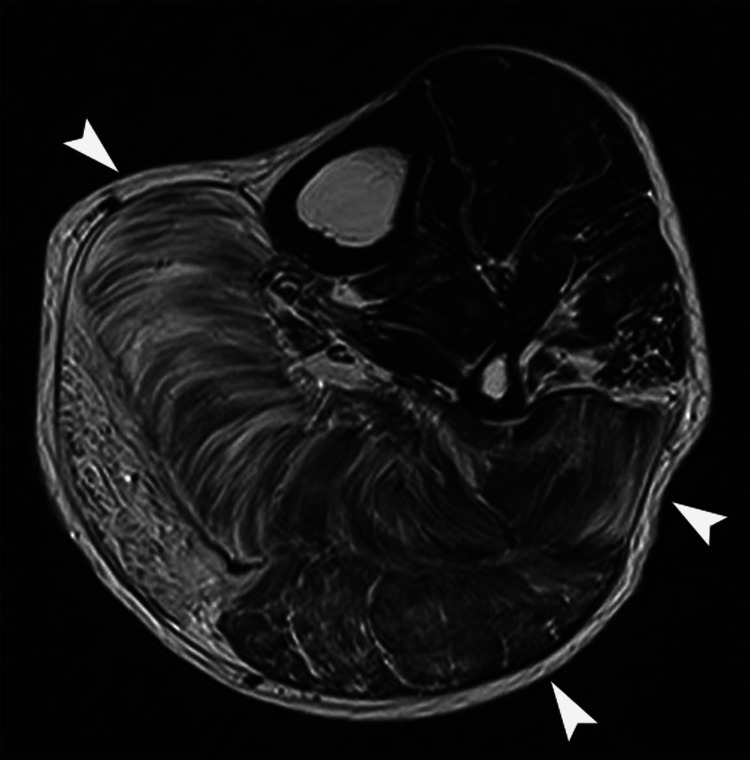
A patient with hereditary advanced limb-girdle muscular dystrophy and neuropathy. Axial T2-weighted MR image of the calf shows diffuse abnormal high signal intensity in enlarged soleus and gastrocnemius muscles (arrowheads), reflecting gross fatty replacement.

Atrophy and fibrofatty infiltration may result in muscle contraction and stiffness, which may predispose weakened muscles to further injury during muscle exertion. On the contrary, some inherited myopathies (metabolic myopathy, dystrophinopathies, sarcoglycanopathies) may manifest with local or generalized muscle hypertrophy or pseudohypertrophy (Figure 8).

Intramuscular fatty infiltration in these myopathies (i.e., Pompe’s disease) may assume a characteristic distribution with selective sparing of specific muscle groups. The presence of fat tissue which is hyperintense to muscle and shows decreased signal intensity on the dedicated fat-suppressed MR images is typically straightforward for abnormal fatty infiltration.

Mass and Mass-Like Lesion Pattern

**Figure 9:**
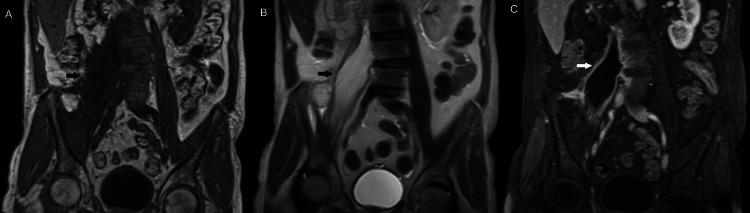
A 69-year-old diabetic man with pyelonephritis. (A-C) Coronal T1- (A), T2- (B), and enhanced, fat-saturated T1-weighted (C) MR images display intramuscular abscess (arrow) of the psoas muscle.

**Figure 10:**
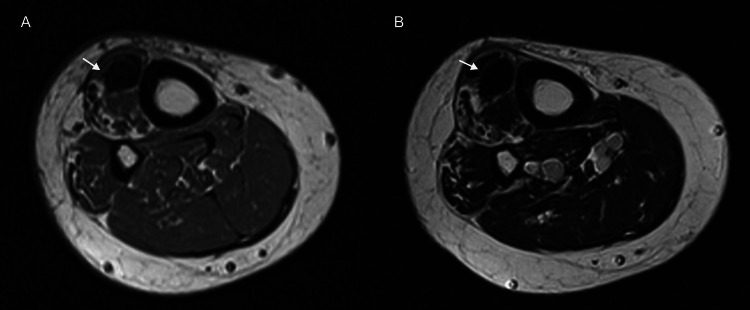
A 36-year-old female rock climber with painful swelling of the proximal leg. (A) and (B) Axial T1- (A) and T2- (B) weighted MR images reveal homogenous, low signal intensity mass in the anterior tibialis muscle, consistent with myositis ossificans.

**Figure 11:**
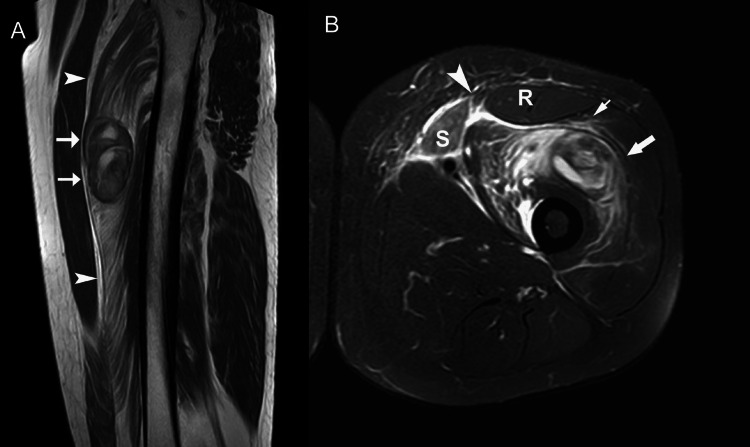
A 27-year-old male with a laceration anterior to the thigh. (A) Sagittal T1-weighted MR image through the thigh shows acute hematoma (arrows) with intermediate signal intensity in injured vastus intermedius muscle. Note the extensive hemorrhage infiltrating the muscle adjacent to hematoma (arrowheads). (B) Axial fat-suppressed fast spin-echo T2-weighted MR image reveals defect (arrowhead) in anterior musculature between sartorius (S) and rectus femoris (R) overlying hematoma (arrow) within enlarged vastus. Findings are consistent with muscle laceration with the formation of hematoma from a stab wound. Note perifascial edema (thin arrow).

**Figure 12:**
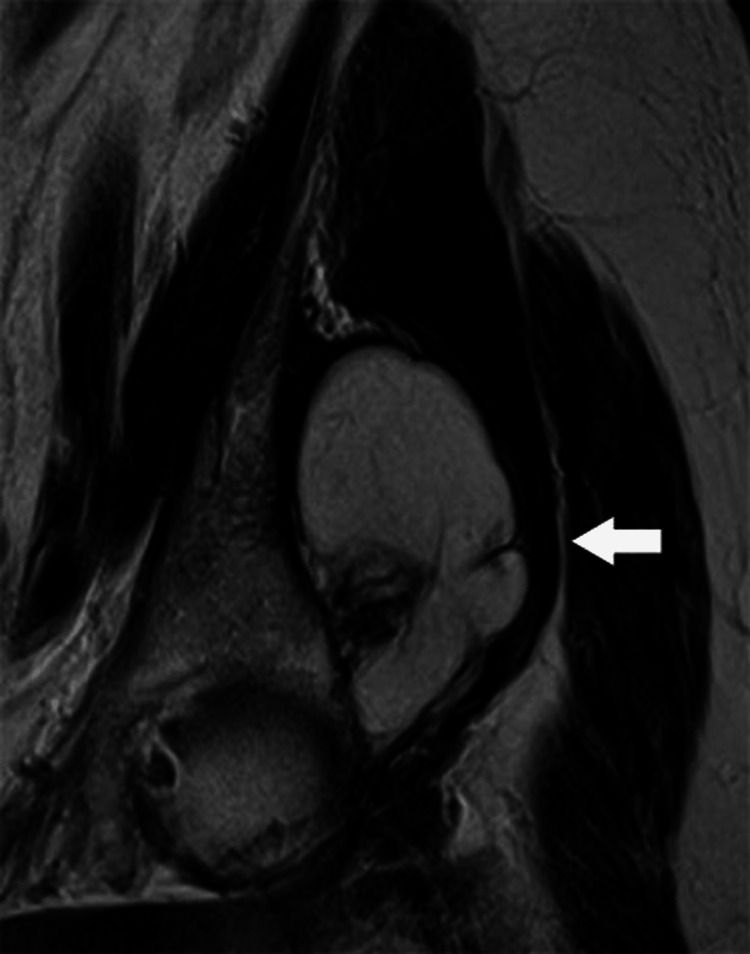
A 60-year-old female with hip pain and inguinal swelling. Coronal T2-weighted MR image shows lipoma (arrow) of arising within the gluteus minimus muscle.

**Figure 13:**
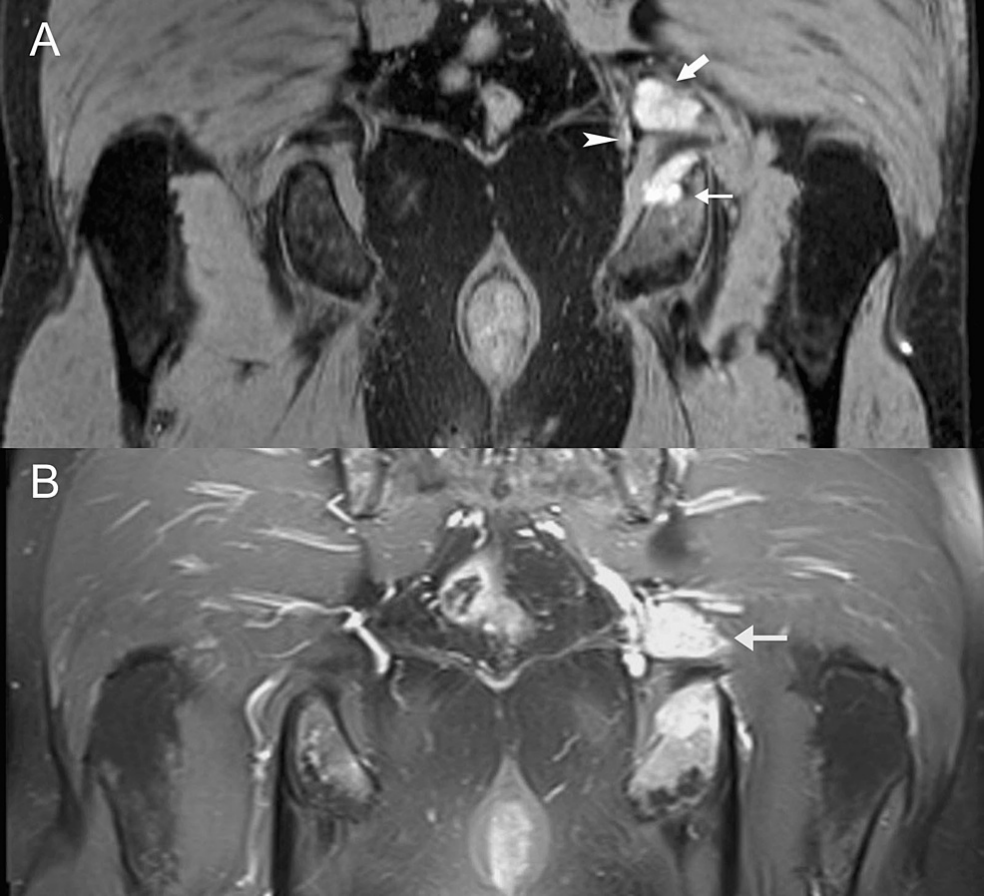
A 58-year-old man with advanced metastatic disease due to the spread of soft-tissue sarcoma. (A) Coronal high-resolution T2-weighted MR image shows infiltration of the bone (ischium) (thin arrow) and the piriformis muscle (arrow).  Note the descending sciatic nerve (arrowhead) adjacent to infiltrated muscle, responsible for sciatica. (B) Coronal T1-weighted MR image with fat saturation after the administration of gadolinium-containing contrast agent depicts ample enhancement of metastatic deposits in both the muscle (arrow) and bone. Abnormal enhancement of the sciatic nerve is also seen.

The mass lesion pattern implies the presence of a space-occupying lesion in muscle. The MR imaging characteristics of lesions producing a mass effect are variable and typically differ remarkably from those of normal muscle on all pulse sequences. Infection (i.e., pyomyositis, abscess, parasitic infection, myositis ossificans), traumatic injury (i.e., hematoma), myonecrosis, muscular sarcoidosis, primary benign or malignant neoplasms arising in muscle (i.e., lipoma, liposarcoma, leiomyosarcoma), and soft-tissue metastases are all associated with an intramuscular mass lesion [19,21-26] (Figures 9-13).

As a general rule, discrete mass lesions that disrupt normal muscle architecture indicate an aggressive or recurrent tumor with an ominous prognosis. MR imaging may afford a detailed analysis of the consistency of a mass lesion that reflects the histology of a given pathologic process. In this regard, MR imaging may definitely enable a thorough pre-biopsy investigation of the nature of a mass or mass-like lesion providing meaningful clues to diagnosis.

Imaging differential diagnosis

Within the broad spectrum of myopathies, knowledge of the characteristic MR imaging patterns of muscle disease is a requisite for maximizing diagnostic accuracy [27]. Although these patterns may be diagnostic in the appropriate clinical context, additional MR imaging alterations in muscle may include abnormal low signal intensity on T2-weighted images, representing calcification, fibrosis, hemosiderin deposition, gas, and foreign bodies. The presence of methemoglobin in muscle, proteinaceous material, melanin, or gadolinium-based contrast material may account for high T1 signal intensity in muscle [28].

Because the potential causes for abnormal signal intensity in muscle are diverse, recognition of the three basic patterns may help narrow the differential diagnosis or suggest a specific diagnosis. The “muscle edema pattern” is the most common pattern of altered muscle signal intensity and may be observed with trauma, rhabdomyolysis, vascular insults, subacute denervation, and infectious/inflammatory myopathies. The “fatty infiltration pattern” is often associated with atrophy and can be seen in cases of hereditary myopathy, chronic muscle injury, disuse, and corticosteroid use. The “mass lesion pattern” is commonly seen with neoplasms, infection, traumatic injuries (myositis ossificans), and muscular sarcoidosis. Assignment of a muscle disorder to one of the three major MR imaging-based patterns may simplify the diagnostic approach for myopathy. However, it should be emphasized that in some instances, the MR imaging findings of muscle disease may reflect the underlying gross pathologic changes rather than provide features for specific imaging diagnosis, and, as such, a wide range of clinical differential diagnoses may eventually need to be entertained.

## Conclusions

Imaging can provide clinically relevant information that may have an impact on therapy and treatment decisions of muscle nosology. In doing so, MR imaging is invaluable in identifying the presence, location, extent, and severity of myopathy.

What we can conclude with certainty is that classification of myopathy into three major patterns based on MR imaging features is important for diagnosis. Indeed, imaging characterization of myopathy may prove useful for addressing the challenges and questions of daily practice, especially in ambiguous clinical cases.

## References

[1] Janssen I, Heymsfield SB, Wang ZM, Ross R (2000). Skeletal muscle mass and distribution in 468 men and women aged 18-88 yr. J Appl Physiol (1985).

[2] Santilli V, Bernetti A, Mangone M, Paoloni M (2014). Clinical definition of sarcopenia. Clin Cases Miner Bone Metab.

[3] Caetano AP, Alves P (2019). Advanced MRI patterns of muscle disease in inherited and acquired myopathies: what the radiologist should know. Semin Musculoskelet Radiol.

[4] Theodorou DJ, Theodorou SJ, Kakitsubata Y (2012). Soft tissue: magnetic resonance imaging findings of myopathies. Soft Tissue: Composition, Mechanisms of Injury and Repair.

[5] Theodorou DJ, Theodorou SJ, Kakitsubata Y (2012). Skeletal muscle disease: patterns of MRI appearances. Br J Radiol.

[6] May DA, Disler DG, Jones EA, Balkissoon AA, Manaster BJ (2000). Abnormal signal intensity in skeletal muscle at MR imaging: patterns, pearls, and pitfalls. Radiographics.

[7] Theodorou SJ, Theodorou DJ, Resnick D (2007). MR imaging findings of pyogenic bacterial myositis (pyomyositis) in patients with local muscle trauma: illustrative cases. Emerg Radiol.

[8] Kransdorf MJ, Murphey MD (2016). Imaging of soft-tissue musculoskeletal masses: fundamental concepts. Radiographics.

[9] Lin J, Jacobson JA, Fessell DP, Weadock WJ, Hayes CW (2000). An illustrated tutorial of musculoskeletal sonography: part 4, musculoskeletal masses, sonographically guided interventions, and miscellaneous topics. AJR Am J Roentgenol.

[10] Ten Dam L, van der Kooi AJ, Verhamme C, Wattjes MP, de Visser M (2016). Muscle imaging in inherited and acquired muscle diseases. Eur J Neurol.

[11] Ortolan P, Zanato R, Coran A, Beltrame V, Stramare R (2015). Role of radiologic imaging in genetic and acquired neuromuscular disorders. Eur J Transl Myol.

[12] Sookhoo S, Mackinnon I, Bushby K, Chinnery PF, Birchall D (2007). MRI for the demonstration of subclinical muscle involvement in muscular dystrophy. Clin Radiol.

[13] Kalia V, Leung DG, Sneag DB, Del Grande F, Carrino JA (2017). Advanced MRI techniques for muscle imaging. Semin Musculoskelet Radiol.

[14] Kim HK, Lindquist DM, Serai SD (2013). Magnetic resonance imaging of pediatric muscular disorders: recent advances and clinical applications. Radiol Clin North Am.

[15] Paoletti M, Pichiecchio A, Cotti Piccinelli S, Tasca G, Berardinelli AL, Padovani A, Filosto M (2019). Advances in quantitative imaging of genetic and acquired myopathies: clinical applications and perspectives. Front Neurol.

[16] Broski SM, Tiegs Heiden CA, Ringler MD (2017). Muscle: ischemia, infarction, and compartment syndrome. Semin Musculoskelet Radiol.

[17] Dimmick S, Linklater JM (2017). Imaging of acute hamstring muscle strain injuries. Semin Musculoskelet Radiol.

[18] Mancillas-Adame LG, González-González JG, Jáquez-Quintana JO, Cardoza-Torres MA, García Ade L (2009). Diabetic myonecrosis in a patient with hepatic cirrhosis: a case report and review of the literature. J Med Case Rep.

[19] Theodorou SJ, Theodorou DJ, Resnick D (2008). Imaging findings of complications affecting the upper extremity in intravenous drug users: featured cases. Emerg Radiol.

[20] Theodorou DJ, Theodorou SJ, Axiotis A, Gianniki M, Tsifetaki N (2021). COVID-19 vaccine-related myositis. QJM.

[21] Kang CH, Shin MJ, Kim SM, Lee SH, Lee CS (2007). MRI of paraspinal muscles in lumbar degenerative kyphosis patients and control patients with chronic low back pain. Clin Radiol.

[22] Crundwell N, O'Donnell P, Saifuddin A (2007). Non-neoplastic conditions presenting as soft-tissue tumours. Clin Radiol.

[23] Koyama T, Ueda H, Togashi K, Umeoka S, Kataoka M, Nagai S (2004). Radiologic manifestations of sarcoidosis in various organs. Radiographics.

[24] McCarthy EF, Sundaram M (2005). Heterotopic ossification: a review. Skeletal Radiol.

[25] Moore SL, Teirstein AE (2003). Musculoskeletal sarcoidosis: spectrum of appearances at MR imaging. Radiographics.

[26] Nabi G, Gupta NP, Gandhi D (2003). Skeletal muscle metastasis from transitional cell carcinoma of the urinary bladder: clinicoradiological features. Clin Radiol.

[27] Smitaman E, Flores DV, Mejía Gómez C, Pathria MN (2018). MR imaging of atraumatic muscle disorders. Radiographics.

[28] Boutin R (2008). Muscle: radiologic perspective - magnetic resonance imaging of muscle. Magnetic Resonance Imaging in Orthopedic Sports Medicine.

